# Analyzing invariants and employing successive reductions for the extended Kadomtsev Petviashvili equation in (3+1) dimensions

**DOI:** 10.1371/journal.pone.0305177

**Published:** 2024-07-02

**Authors:** Akhtar Hussain, F. D. Zaman, Saud Owyed, Jorge Herrera, Mohammed Sallah

**Affiliations:** 1 Abdus Salam School of Mathematical Sciences, Government College University, Lahore, Pakistan; 2 Mathematics Department, College of Science, University of Bisha, Bisha, Saudi Arabia; 3 Facultad de Ciencias Naturales e Ingenieria, Universidad de Bogota Jorge Tadeo Lozano, Bogota, Colombia; 4 Applied Mathematical Physics Research Group, Physics Department, Faculty of Science, Mansoura University, Mansoura, Egypt; College of Mathematics and Systems Science, Shandong University of Science and Technology, CHINA

## Abstract

In this research, we employ the potent technique of Lie group analysis to derive analytical solutions for the (3+1)-extended Kadomtsev-Petviashvili (3D-EKP) equation. The systematic application of this method enables the identification of Lie point symmetries associated with the equation, leading to the derivation of an optimal system of one-dimensional subalgebras relevant to the equation. This optimal system is utilized to obtain several invariant solutions. The Lie group method is subsequently applied to the reduced governing equations derived from the given equation. We complement our findings with Mathematica simulations illustrating some of the obtained solutions. Furthermore, a direct approach is used to investigate local conservation laws. Importantly, our study addresses a gap in the exploration of the 3D-EXP equation using group theoretic methods, making our findings novel in this context.

## 1 Introduction

In recent times, there has been a notable focus on the study of nonlinear problems, particularly on nonlinear partial differential equations (abbreviated as NLPDEs) and their precise traveling wave solutions [1–3]. This heightened attention is motivated by the recognition that numerous physical phenomena can be accurately described by these NLPDEs. The captivating aspect of nonlinearity has attracted numerous scientists who view nonlinear science as the primary frontier for gaining a deep understanding of nature. Researchers have explored various models in this field, including the study of the modified Kadomtsev-Petviashvili-Burgers (KP-Burgers) type equations, as examined in [4]. This research contributes to our knowledge of complex phenomena in physics and fluid mechanics, providing insights into fundamental aspects of nature and potential practical applications. The generalized Korteweg-de Vries-Zakharov-Kuznetsov equation is a mathematical model that has been investigated in reference [5]. This equation describes cold immobile background species, mixtures of warm adiabatic fluid, and hot isothermal fluid, which are relevant to fluid dynamics. Additionally, reference [6] discusses the generalized and modified Zakharov-Kuznetsov model, which was employed to study ion-acoustic drift solitary waves in a magnetoplasma containing electron-positron-ion particles. Moreover, in reference [7], the emphasis was on exploring the properties and interactions of bright solitons within the Fokas-Lenells system. This system represents the behavior of femtosecond optical pulses in birefringent optical fibers. The study aimed to gain insights into the characteristics of these solitons and their interactions, contributing to our understanding of femtosecond optical pulse propagation in birefringent fibers, which is relevant to optical communication and fiber optics. Moreover, [8] investigated the Boussinesq-Burgers-type equations that describe shallow water waves near lakes or ocean beaches. These examples represent only a fraction of the research conducted in this field, and for more information, please refer to [9–17].

A wide range of partial differential equations (PDEs) can describe nonlinear wave motion. One of these equations, known as the Kadomtsev-Petviashvili equation (abbreviated as KP), is extensively studied in the field of mathematical physics. Investigating nonlinear waves is of great importance in understanding various nonlinear phenomena. Researchers have examined different versions of the KP equation, and one such version is the three-dimensional Kadomtsev-Petviashvili equation, as documented in [18]

$$\begin{array}{c} {\left(U_{t}+6UU_{x}+U_{xxx}\right)}_{x}\pm 3U_{yy}\pm 3U_{zz}=0. \end{array}$$ (1)

The equation mentioned in the paragraph can be traced back to the influential work conducted by Soviet physicists Kadomtsev and Petviashvili in 1970 [19]. Eq (1) characterizes wave behavior in a scenario where the water wavelength to water depth ratio is extremely small, and the influence of nonlinear restoring forces is negligible. The equation being discussed is an extended form of the Korteweg-de Vries (abbreviated as KdV) equation, originally named after Korteweg and De Vries [20], a Dutch mathematician. In Eq (1), the term *UU*_*x*_ corresponds to the nonlinear component of the wave equation. The last two terms in the equation account for diffractive divergence, while the highest-order term represents weak dispersion [21]. The ± sign attached to the last two terms in the equation typically indicates the two possible directions of dispersion. It signifies that dispersion can occur in both negative and positive directions along the relevant coordinate axis. Since its inception, numerous researchers have investigated Eq (1), as documented in [22]. These studies encompass a wide range of analyses, including Painlevé analysis [23], establishing closed-form multiple wave solutions [24], the establishment of soliton stability properties [25], and the determination of integrability features [26].

In this study, our objective is to explore the group theoretic technique associated with the 3D-EKP equation. The equation is given by [27]

$$\begin{array}{c} U_{tx}+\frac{3}{2}U^{3}U_{x}+\frac{3}{2}U^{2}U_{xx}+3UU_{x}^{2}+3U_{x}U_{xx}+U_{yy}+U_{zz}=0. \end{array}$$ (2)

Lü *et al.* [27] derived this equation using generalized bilinear operators associated with the prime number *p* = 3. They employed the concept of an extended Kadomtsev-Petviashvili-like (3D-EKP) equation to characterize it. Through symbolic computation, they generated eighteen classes of rational solutions for the resulting 3D-EKP equation by exploring polynomial solutions to the corresponding generalized bilinear equation. Additionally, Yu and Sun [28] constructed lump solutions, rationally localized in all spatial directions, for its two-dimensionally reduced cases using a generalized bilinear differential equation method. Notably, this equation finds numerous applications in wave theory, mathematical physics, and engineering sciences.

The main objective of this article is to perform a Lie group analysis of the 3D-EKP Eq (2). Initially, we apply the Lie group method to derive the eight-dimensional Lie algebra, utilizing it to discuss the optimal system of one-dimensional subalgebras. This system proves valuable in obtaining all possible non-similar vector fields, crucial for deriving invariant solutions. Our motivation extends to exploring local conservation laws for the 3D-EKP Eq (2) through the multiplier approach. Lie group analysis stands as a valuable technique for handling invariant solutions and conservation laws in nonlinear differential equations, particularly in the realm of NLPDEs. This method leverages group-theoretic structures, such as a system of subalgebras, to address invariant solutions. Numerous studies in the literature have tackled the Lie group analysis of NLPDEs [29–34], among others. Building upon these references, our study aims to fill the research gap concerning group-theoretic properties of the 3D-EKP Eq (2). It explores a significant class of solutions novel invariant solutions characterized by invariance under specific Lie symmetry generators. Additionally, our study breaks new ground as none of the existing literature delves into the conservation laws of the 3D-EKP Eq (2).

The article is organized as follows: Section 2 explains the Lie symmetry method applied to the 3D-EKP Eq (2). Section 3 discusses the symmetry reductions of the 3D-EKP Eq (2), focusing on the reduction of dimensions and simplification of the equation. Section 4 delves into the discussion of local conservation laws utilizing the multiplier method for the 3D-EKP Eq (2). Section 5 focuses on the graphical interpretation of the obtained solutions. Finally, in Section 6, the article concludes by highlighting future research directions and potential areas of further investigation.

## 2 Symmetry generators and optimal system for 3D-EKP Eq (2)

Within this section, our focus lies in the exploration of Lie symmetries and the identification of an optimal system for Eq (2). We undertake this inquiry by postulating a one-parameter Lie group of transformations, as delineated in prior work [35]

$$\begin{array}{c} \begin{array}{c} \bar{x}\to x+\varsigma \Phi^{1}\left(x,y,z,t,U\right)+O\left(\varsigma^{2}\right),\\ \bar{y}\to y+\varsigma \Phi^{2}\left(x,y,z,t,U\right)+O\left(\varsigma^{2}\right),\\ \bar{z}\to z+\varsigma \Phi^{3}\left(x,y,z,t,U\right)+O\left(\varsigma^{2}\right),\\ \bar{t}\to t+\varsigma \Phi^{4}\left(x,y,z,t,U\right)+O\left(\varsigma^{2}\right),\\ \bar{U}\to U+\varsigma \varrho \left(x,y,z,t,U\right)+O\left(\varsigma^{2}\right). \end{array} \end{array}$$ (3)

The infinitesimal operator linked to the transformations above is given by [36]

$$\begin{array}{c} B=\Phi^{1}\frac{\partial }{\partial x}+\Phi^{2}\frac{\partial }{\partial y}+\Phi^{3}\frac{\partial }{\partial z}+\Phi^{4}\frac{\partial }{\partial t}+\varrho \frac{\partial }{\partial U}\cdot \end{array}$$ (4)

The task is to discover the coefficient functions Φ^1^, Φ^2^, Φ^3^, Φ^4^ and *ϱ*, while ensuring that the operator *B* satisfies the symmetry condition [37]

$$\begin{array}{c} B^{\left[2\right]}\left(U_{tx}+\frac{3}{2}U^{3}U_{x}+\frac{3}{2}U^{2}U_{xx}+3UU_{x}^{2}+3U_{x}U_{xx}+U_{yy}+U_{zz}\right){|}_{\left(2\right)}=0, \end{array}$$ (5)

where *B*^[2]^ is the second prolongation of *B*. The invariance condition for the Eq (2) becomes

$$\begin{array}{c} B+\varrho^{t}\partial_{U_{t}}+\varrho^{x}\partial_{U_{x}}+\varrho^{tx}\partial_{U_{tx}}+\varrho^{xx}\partial_{U_{xx}}+\varrho^{yy}\partial_{U_{yy}}+\varrho^{zz}\partial_{U_{zz}}=0. \end{array}$$ (6)

The values of *ϱ*^*x*^, *ϱ*^*tx*^, *ϱ*^*xx*^, *ϱ*^*yy*^, and *ϱ*^*zz*^ in this scenario are determined using the general relations provided by

$$\begin{array}{cc} \varrho^{i} & =D_{i}\left(\varrho \right)-U_{j}D_{i}(\Phi^{j}),\\ \varrho^{ij} & =D_{j}(\varrho^{i})-U_{ik}D_{j}(\Phi^{k}),i,j,k=1,2,3,4. \end{array}$$ (7)

The coefficient functions in above equation, along with the total derivatives *D*_*i*_ in (7), are also expressed in a general form as follows

$$D_{i}=\frac{\partial }{\partial x^{i}}+U_{i}\frac{\partial }{\partial U}+U_{ij}\frac{\partial }{\partial U_{j}}+\cdots ,$$

which can also be used for *D*_*j*_. Thus, inserting the values in Eq (6) and comparing different powers, we get

$$\begin{array}{c} \varrho_{xU}=0,\;\Phi_{x}^{3}=0,\;\Phi_{x}^{2}=0,\;\Phi_{x}^{4}=0,\;\Phi_{y}^{4}=0,\;\Phi_{z}^{4}=0,\;\varrho_{UU}=0,\;\Phi_{U}^{3}=0,\\ \Phi_{U}^{2}=0,\;\Phi_{U}^{1}=0,\;\Phi_{U}^{4}=0,\;\Phi_{z}^{2}+\Phi_{y}^{3}=0,\;2\Phi_{z}^{1}+\Phi_{t}^{3}=0,\;2\Phi_{y}^{1}+\Phi_{t}^{2}=0,\\ \Phi_{xx}^{1}-U\Phi_{x}^{1}-\varrho =0,\;\varrho_{U}-2\Phi_{x}^{1}+\Phi_{t}^{4}=0,\;2\varrho_{yU}+\Phi_{yz}^{3}-\Phi_{yy}^{2}=0,\\ 2\Phi_{y}^{2}-\Phi_{x}^{1}-\Phi_{t}^{4}=0,\;2\Phi_{z}^{3}-\Phi_{x}^{1}-\Phi_{t}^{4}=0,\;2\varrho_{zU}-\Phi_{zz}^{3}-\Phi_{yy}^{3}=0,\\ 3\Phi_{x}^{1}U^{2}-3\Phi_{t}^{4}U^{2}-6\varrho U-6\varrho_{x}+2\Phi_{t}^{1}=0,\\ 3\varrho_{x}U^{3}+3\varrho_{xx}U^{2}+2\varrho_{zz}+2\varrho_{yy}+2\varrho_{tx}=0,\\ 3\Phi_{t}^{4}U^{3}+9\varrho U^{2}-3\Phi_{tx}^{4}U^{2}+6\varrho_{x}U+2\varrho_{tU}+\Phi_{tz}^{3}+\Phi_{ty}^{2}=0. \end{array}$$ (8)

By solving the system in Eq (8), we get the following infinitesimals

$$\begin{array}{c} \Phi^{1}=\frac{1}{2}(2c_{2}+c_{6}x-c_{4}y-c_{8}z),\;\Phi^{2}=c_{3}+c_{4}t+c_{6}y-c_{7}z,\\ \Phi^{3}=c_{5}+c_{8}t+c_{7}y+c_{6}z,\;\Phi^{4}=c_{1}+\frac{3c_{6}t}{2}\;\varrho =-\frac{c_{6}U}{2}. \end{array}$$

These formulas result in eight symmetry generators, which are expressed as follows,

$$\begin{array}{c} \begin{array}{cc} & B_{1}=\frac{\partial }{\partial t},\;B_{2}=\frac{\partial }{\partial x},\;B_{3}=\frac{\partial }{\partial y},\;B_{4}=\frac{\partial }{\partial z},\;B_{5}=2t\frac{\partial }{\partial y}-y\frac{\partial }{\partial x},\\ & B_{6}=2t\frac{\partial }{\partial z}-z\frac{\partial }{\partial x},\;B_{7}=y\frac{\partial }{\partial z}-z\frac{\partial }{\partial y},\;B_{8}=3t\frac{\partial }{\partial t}+x\frac{\partial }{\partial x}+2y\frac{\partial }{\partial y}+2z\frac{\partial }{\partial z}-U\frac{\partial }{\partial U}\cdot \end{array} \end{array}$$ (9)

The adjoint representation is defined as [31]

$$\begin{array}{c} Ad\left(\text{exp}\;\left(\varsigma B_{i}\right).B_{j}\right)=B_{j}-\varsigma \left[B_{i},B_{j}\right]+\frac{\varsigma^{2}}{2!}\left[B_{i},\left[B_{i},B_{j}\right]\right]-\cdots \end{array}$$ (10)

where [*B*_*i*_, *B*_*j*_] is the commutator for the Lie algebra defined as,

$$\begin{array}{c} \left[B_{m},B_{n}\right]=B_{m}B_{n}-B_{n}B_{m}. \end{array}$$ (11)

*ς* is a parameter. Both the commutator relation and adjoint representation for the Lie algebra (9) are given in Tables (1)–(3).

**Table 1:** Commutator table.

[*B*_*i*_, *B*_*j*_]	*B*_1_	*B*_2_	*B*_3_	*B*_4_	*B*_5_	*B*_6_	*B*_7_	*B*_8_
*B*_1_	0	0	0	0	2*B*_3_	2*B*_4_	0	3*B*_1_
*B*_2_	0	0	0	0	0	0	0	*B*_2_
*B*_3_	0	0	0	0	−*B*_2_	0	*B*_4_	2*B*_3_
*B*_4_	0	0	0	0	0	−*B*_2_	−*B*_3_	2*B*_4_
*B*_5_	−2*B*_3_	0	*B*_2_	0	0	0	*B*_6_	−*B*_5_
*B*_6_	−2*B*_4_	0	0	*B*_2_	0	0	−*B*_5_	−*B*_6_
*B*_7_	0	0	−*B*_4_	*B*_3_	−*B*_6_	*B*_5_	0	0
*B*_8_	−3*B*_1_	−*B*_2_	−2*B*_3_	−2*B*_4_	*B*_5_	*B*_6_	0	0

**Table 2:** Adjoint table.

*Ad*(*e*^*ς*^)	*B*_1_	*B*_2_	*B*_3_	*B*_4_
*B*_1_	*B*_1_	*B*_2_	*B*_3_	*B*_4_
*B*_2_	*B*_1_	*B*_2_	*B*_3_	*B*_4_
*B*_3_	*B*_1_	*B*_2_	*B*_3_	*B*_4_
*B*_4_	*B*_1_	*B*_2_	*B*_3_	*B*_4_
*B*_5_	*B*_1_ + 2*ςB*_3_ − *ς*^2^*B*_2_	*B*_2_	*B*_3_ − *ςB*_2_	*B*_4_
*B*_6_	*B*_1_ + 2*ςB*_4_ − *ς*^2^*B*_2_	*B*_2_	*B*_3_	*B*_4_ − *ςB*_2_
*B*_7_	*B*_1_	*B*_2_	cos *ςB*_3_ + sin *ςB*_4_	− sin *ςB*_3_ + cos *ςB*_4_
*B*_8_	*e* ^3*ς*^ *B*_1_	*e* ^*ς*^ *B*_2_	*e* ^2*ς*^ *B*_3_	*e* ^2*ς*^ *B*_4_

**Table 3:** Adjoint table.

*Ad*(*e*^*ς*^)	*B*_5_	*B*_6_	*B*_7_	*B*_8_
*B*_1_	*B*_5_ − 2*ςB*_3_	*B*_6_ − 2*ςB*_4_	*B*_7_	*B*_8_ − 3*ςB*_1_
*B*_2_	*B*_5_	*B*_6_	*B*_7_	*B*_8_ − *ςB*_2_
*B*_3_	*B*_5_ + *ςB*_2_	*B*_6_	*B*_7_ − *ςB*_4_	*B*_8_ − 2*ςB*_3_
*B*_4_	*B*_5_	*B*_6_ + *ςB*_2_	*B*_7_ + *ςB*_3_	*B*_8_ − 2*ςB*_4_
*B*_5_	*B*_5_	*B*_6_	*B*_7_ − *ςB*_6_	*B*_8_ + *ςB*_5_
*B*_6_	*B*_5_	*B*_6_	*B*_7_ + *ςB*_5_	*B*_8_ + *ςB*_6_
*B*_7_	cos *ςB*_5_ + sin *ςB*_6_	− sin *ςB*_5_ + cos *ςB*_6_	*B*_7_	*B*_8_
*B*_8_	*e* ^−*ς*^ *B*_5_	*e* ^−*ς*^ *B*_6_	*B*_7_	*B*_8_

### 2.1 Optimal system [31]

Consider a general element $B\in L_{8}.$ We have,

$$\begin{array}{c} B=q_{1}B_{1}+q_{2}B_{2}+q_{3}B_{3}+q_{4}B_{4}+q_{5}B_{5}+q_{6}B_{6}+q_{7}B_{7}+q_{8}B_{8}. \end{array}$$ (12)

**Case 1**: *q*_8_ ≠ 0. Then we have,

$$\begin{array}{c} B=q_{1}B_{1}+q_{2}B_{2}+q_{3}B_{3}+q_{4}B_{4}+q_{5}B_{5}+q_{6}B_{6}+q_{7}B_{7}+q_{8}B_{8} \end{array}$$ (13)

$$\begin{array}{c} B^{\prime }=Ad\left(e^{\varsigma }B_{5}\right)B=q_{1}B_{1}+q_{4}B_{4}+q_{7}B_{7}+q_{8}B_{8} \end{array}$$ (14)

$$\begin{array}{c} B^{\prime \prime }=Ad\left(e^{\varsigma }B_{1}\right)B^{\prime }=q_{4}B_{4}+q_{7}B_{7}+q_{8}B_{8} \end{array}$$ (15)

$$\begin{array}{c} B^{\prime \prime \prime }=Ad\left(e^{\varsigma }B_{3}\right)B^{\prime \prime }=q_{7}B_{7}+q_{8}B_{8}. \end{array}$$ (16)

Thus, Ω_1_ = *B*_7_ + *cB*_8_, *c* ≠ 0.

**Case 2**: *q*_8_ ≠ 0, *q*_7_ = 0. Then we have,

$$\begin{array}{c} B=q_{1}B_{1}+q_{2}B_{2}+q_{3}B_{3}+q_{4}B_{4}+q_{5}B_{5}+q_{6}B_{6}+q_{8}B_{8} \end{array}$$ (17)

$$\begin{array}{c} B^{\prime }=Ad\left(e^{\varsigma }B_{5}\right)B=q_{1}B_{1}+q_{4}B_{4}+q_{6}B_{6}+q_{8}B_{8} \end{array}$$ (18)

$$\begin{array}{c} B^{\prime \prime }=Ad\left(e^{\varsigma }B_{1}\right)B^{\prime }=q_{6}B_{6}+q_{8}B_{8} \end{array}$$ (19)

$$\begin{array}{c} B^{\prime \prime \prime }=Ad\left(e^{\varsigma }B_{6}\right)B^{\prime \prime }=B_{8}, \end{array}$$ (20)

Thus, Ω_2_ = *B*_8_.

**Case 3**: *q*_8_ = 0, *q*_7_ ≠ 0. Then we have,

$$\begin{array}{c} B=q_{1}B_{1}+q_{2}B_{2}+q_{3}B_{3}+q_{4}B_{4}+q_{5}B_{5}+q_{6}B_{6}+q_{7}B_{7} \end{array}$$ (21)

$$\begin{array}{c} B^{\prime }=Ad\left(e^{\varsigma }B_{5}\right)B=q_{1}B_{1}+q_{4}B_{4}+q_{5}B_{5}+q_{7}B_{7} \end{array}$$ (22)

$$\begin{array}{c} B^{\prime \prime }=Ad\left(e^{\varsigma }B_{6}\right)B^{\prime }=q_{1}B_{1}+q_{7}B_{7} \end{array}$$ (23)

$$\begin{array}{c} B^{\prime \prime \prime }=Ad\left(e^{\varsigma }B_{8}\right)B^{\prime \prime }=q_{1}B_{1}+e^{-3\varsigma }q_{7}B_{7}. \end{array}$$ (24)

Thus, Ω_3_ = *B*_1_ ± *B*_7_.

**Case 4**: *q*_8_ = 0, *q*_7_ ≠ 0, *q*_1_ = 0. Then we have,

$$\begin{array}{c} B=q_{2}B_{2}+q_{3}B_{3}+q_{4}B_{4}+q_{5}B_{5}+q_{6}B_{6}+q_{7}B_{7} \end{array}$$ (25)

$$\begin{array}{c} B^{\prime }=Ad\left(e^{\varsigma }B_{4}\right)B=q_{4}B_{4}+q_{5}B_{5}+q_{6}B_{6}+q_{7}B_{7} \end{array}$$ (26)

$$\begin{array}{c} B^{\prime \prime }=Ad\left(e^{\varsigma }B_{1}\right)B^{\prime }=q_{5}B_{5}+q_{6}B_{6}+q_{7}B_{7} \end{array}$$ (27)

$$\begin{array}{c} B^{\prime \prime \prime }=Ad\left(e^{\varsigma }B_{6}\right)B^{\prime \prime }=q_{6}B_{6}+q_{7}B_{7} \end{array}$$ (28)

$$\begin{array}{c} B^{\prime \prime \prime \prime }=Ad\left(e^{\varsigma }B_{5}\right)B^{\prime \prime \prime }=q_{7}B_{7}. \end{array}$$ (29)

Thus, Ω_4_ = *B*_7_.

**Case 5**: *q*_6_ = 0, *q*_8_ = 0, *q*_5_ = 0, *q*_4_ = 0, *q*_3_ = 0, *q*_1_ = 0. Then we have,

$$\begin{array}{c} B=q_{2}B_{2}+q_{7}B_{7} \end{array}$$ (30)

$$\begin{array}{c} B^{\prime }=Ad\left(e^{\varsigma }B_{8}\right)B=q_{2}B_{2}+e^{-\varsigma }q_{7}B_{7}. \end{array}$$ (31)

Thus, Ω_5_ = *B*_2_ ± *B*_7_.

**Case 6**: *q*_8_ = 0, *q*_7_ = 0, *q*_6_ ≠ 0, *q*_5_ = 0. Then we have,

$$\begin{array}{c} B=q_{1}B_{1}+q_{2}B_{2}+q_{3}B_{3}+q_{4}B_{4}+q_{6}B_{6} \end{array}$$ (32)

$$\begin{array}{c} B^{\prime }=Ad\left(e^{\varsigma }B_{5}\right)B=q_{1}B_{1}+q_{4}B_{4}+q_{6}B_{6} \end{array}$$ (33)

$$\begin{array}{c} B^{\prime \prime }=Ad\left(e^{\varsigma }B_{1}\right)B^{\prime }=q_{1}B_{1}+q_{6}B_{6} \end{array}$$ (34)

$$\begin{array}{c} B^{\prime \prime \prime }=Ad\left(e^{\varsigma }B_{8}\right)B^{\prime \prime }=q_{1}B_{1}+e^{-4\varsigma }q_{6}B_{6}. \end{array}$$ (35)

Thus, Ω_6_ = *B*_1_ ± *B*_6_.

**Case 7**: *q*_7_ = 0, *q*_8_ = 0, *q*_6_ ≠ 0, *q*_5_ = 0, *q*_1_ = 0. Then we have,

$$\begin{array}{c} B=q_{2}B_{2}+q_{3}B_{3}+q_{4}B_{4}+q_{6}B_{6} \end{array}$$ (36)

$$\begin{array}{c} B^{\prime }=Ad\left(e^{\varsigma }B_{6}\right)B=q_{3}B_{3}+q_{4}B_{4}+q_{6}B_{6} \end{array}$$ (37)

$$\begin{array}{c} B^{\prime \prime }=Ad\left(e^{\varsigma }B_{1}\right)B^{\prime }=q_{3}B_{3}+q_{6}B_{6} \end{array}$$ (38)

$$\begin{array}{c} B^{\prime \prime \prime }=Ad\left(e^{\varsigma }B_{8}\right)B^{\prime \prime }=q_{3}B_{3}+e^{-3\varsigma }q_{6}B_{6}. \end{array}$$ (39)

Thus, Ω_7_ = *B*_3_ ± *B*_6_.

**Case 8**: *q*_8_ = 0, *q*_7_ = 0, *q*_6_ ≠ 0, *q*_5_ = 0, *q*_1_ = 0, *q*_3_ = 0. Then we have,

$$\begin{array}{c} B=q_{2}B_{2}+q_{4}B_{4}+q_{6}B_{6} \end{array}$$ (40)

$$\begin{array}{c} B^{\prime }=Ad\left(e^{\varsigma }B_{6}\right)B=q_{4}B_{4}+q_{6}B_{6} \end{array}$$ (41)

$$\begin{array}{c} B^{\prime \prime }=Ad\left(e^{\varsigma }B_{1}\right)B^{\prime }=q_{6}B_{6}. \end{array}$$ (42)

Thus, Ω_8_ = *B*_6_.

**Case 9**: *q*_7_ = 0, *q*_8_ = 0, *q*_6_ = 0, *q*_5_ ≠ 0. Then we have,

$$\begin{array}{c} B=q_{1}B_{1}+q_{2}B_{2}+q_{3}B_{3}+q_{4}B_{4}+q_{5}B_{5} \end{array}$$ (43)

$$\begin{array}{c} B^{\prime }=Ad\left(e^{\varsigma }B_{5}\right)B=q_{1}B_{1}+q_{4}B_{4}+q_{5}B_{5} \end{array}$$ (44)

$$\begin{array}{c} B^{\prime \prime }=Ad\left(e^{\varsigma }B_{6}\right)B^{\prime }=q_{1}B_{1}+q_{5}B_{5} \end{array}$$ (45)

$$\begin{array}{c} B^{\prime \prime \prime }=Ad\left(e^{\varsigma }B_{8}\right)B^{\prime \prime }=q_{1}B_{1}+e^{-4\varsigma }q_{5}B_{5}. \end{array}$$ (46)

Thus, Ω_9_ = *B*_1_ ± *B*_5_.

**Case 10**: *q*_7_ = 0, *q*_8_ = 0, *q*_6_ = 0, *q*_5_ ≠ 0, *q*_1_ = 0. Then we have,

$$\begin{array}{c} B=q_{2}B_{2}+q_{3}B_{3}+q_{4}B_{4}+q_{5}B_{5} \end{array}$$ (47)

$$\begin{array}{c} B^{\prime }=Ad\left(e^{\varsigma }B_{1}\right)B=q_{2}B_{2}+q_{4}B_{4}+q_{5}B_{5} \end{array}$$ (48)

$$\begin{array}{c} B^{\prime \prime }=Ad\left(e^{\varsigma }B_{3}\right)B^{\prime }=q_{4}B_{4}+q_{5}B_{5} \end{array}$$ (49)

$$\begin{array}{c} B^{\prime \prime \prime }=Ad\left(e^{\varsigma }B_{8}\right)B^{\prime \prime }=q_{4}B_{4}+e^{-3\varsigma }q_{5}B_{5}. \end{array}$$ (50)

Thus, Ω_10_ = *B*_4_ ± *B*_5_.

**Case 11**: *q*_7_ = 0, *q*_8_ = 0, *q*_6_ = 0, *q*_5_ ≠ 0, *q*_4_ = 0, *q*_1_ = 0. Then we have,

$$\begin{array}{c} B=q_{2}B_{2}+q_{3}B_{3}+q_{5}B_{5} \end{array}$$ (51)

$$\begin{array}{c} B^{\prime }=Ad\left(e^{\varsigma }B_{1}\right)B=q_{2}B_{2}+q_{5}B_{5} \end{array}$$ (52)

$$\begin{array}{c} B^{\prime \prime }=Ad\left(e^{\varsigma }B_{3}\right)B^{\prime }=q_{5}B_{5}. \end{array}$$ (53)

Thus, Ω_11_ = *B*_5_.

**Case 12**: *q*_7_ = 0, *q*_8_ = 0, *q*_6_ = 0, *q*_5_ = 0, *q*_4_ ≠ 0, *q*_3_ = 0, *q*_1_ = 0. Then we have,

$$\begin{array}{c} B=q_{2}B_{2}+q_{4}B_{4} \end{array}$$ (54)

$$\begin{array}{c} B^{\prime }=Ad\left(e^{\varsigma }B_{6}\right)B=q_{4}B_{4}. \end{array}$$ (55)

Thus, Ω_12_ = *B*_4_.

**Case 13**: *q*_7_ = 0, *q*_8_ = 0, *q*_6_ = 0, *q*_5_ = 0, *q*_4_ = 0, *q*_3_ ≠ 0, *q*_1_ = 0. Then we have,

$$\begin{array}{c} B=q_{2}B_{2}+q_{3}B_{3} \end{array}$$ (56)

$$\begin{array}{c} B^{\prime }=Ad\left(e^{\varsigma }B_{5}\right)B=q_{3}B_{3}. \end{array}$$ (57)

Thus, Ω_13_ = *B*_3_.

**Case 14**: *q*_7_ = 0, *q*_8_ = 0, *q*_6_ = 0, *q*_5_ = 0, *q*_4_ = 0, *q*_3_ = 0, *q*_1_ = 0. Then we have,

$$\begin{array}{c} B=q_{2}B_{2}. \end{array}$$ (58)

Thus, Ω_14_ = *B*_2_.

**Case 15**: *q*_7_ = 0, *q*_8_ = 0, *q*_6_ = 0, *q*_5_ = 0, *q*_4_ = 0, *q*_1_ ≠ 0. Then we have,

$$\begin{array}{c} B=q_{1}B_{1}+q_{2}B_{2}+q_{3}B_{3} \end{array}$$ (59)

$$\begin{array}{c} B^{\prime }=Ad\left(e^{\varsigma }B_{5}\right)B=q_{1}B_{1}. \end{array}$$ (60)

Thus, Ω_15_ = *B*_1_.

Therefore, the optimal system for 3D-EKP Eq (2) is expressed as follows

$$\begin{array}{l} \Omega_{1}=B_{7}+cB_{8},c\neq 0\\ \Omega_{2}=B_{8}\\ \Omega_{3}=B_{1}\pm B_{7}\\ \Omega_{4}=B_{7}\\ \Omega_{5}=B_{2}\pm B_{7}\\ \Omega_{6}=B_{1}\pm B_{6}\\ \Omega_{7}=B_{3}\pm B_{6}\\ \Omega_{8}=B_{6} \end{array}\;\;\;|\;\;\;\begin{array}{l} \Omega_{9}=B_{1}\pm B_{5}\\ \Omega_{10}=B_{4}\pm B_{5}\\ \Omega_{11}=B_{5}\\ \Omega_{12}=B_{4}\\ \Omega_{13}=B_{3}\\ \Omega_{14}=B_{2}\\ \Omega_{15}=B_{1}. \end{array}$$

## 3 Group invariant solutions of the 3D-EKP Eq (2)

**Case 1**: Ω_15_ = 〈*B*_1_〉.

The characteristic form for the vector field $B_{1}=\frac{\partial }{\partial t}$ can be formulated as

$$\frac{dU}{0}=\frac{dz}{0}=\frac{dy}{0}=\frac{dx}{0}=\frac{dt}{1},$$

and leaves the invariants (*l*, *m*, *n*) = (*x*, *y*, *z*) by the transformation *U*(*x*, *y*, *z*, *t*) = *f*(*l*, *m*, *n*). Then Eq (2) reduces to (2+1) dimensional nonlinear PDE given by

$$\begin{array}{c} \left(3f^{2}+6f_{l}\right)f_{ll}+3f^{3}f_{l}+6f{f_{l}}^{2}+2f_{mm}+2f_{nn}=0. \end{array}$$ (61)

Once more, the Lie symmetry method is applied to PDE (61), leading to the emergence of novel infinitesimals

$$\begin{array}{cc} \xi_{l} & =\frac{l}{2}c_{2}+c_{4},\\ \xi_{m} & =-c_{1}n+c_{2}m+c_{5},\\ \xi_{n} & =c_{1}m+c_{2}n+c_{3},\\ \eta_{f} & =-\frac{f}{2}c_{2}. \end{array}$$ (62)

**Case 1.1**: Under the condition *c*_5_ = 1 and all other constants set to zero, the characteristic form for (62) manifests as follows

$$\frac{df}{0}=\frac{dl}{0}=\frac{dm}{1}=\frac{dn}{0},$$

and leaves the invariants (*r*, *s*) = (*l*, *n*) by the transformation *f*(*l*, *m*, *n*) = *g*(*r*, *s*). Then Eq (61) reduces to (1+1) dimensional nonlinear PDE given by

$$\begin{array}{c} \left(3g^{2}+6g_{r}\right)g_{rr}+3g^{3}g_{r}+6g{g_{r}}^{2}+2g_{ss}=0. \end{array}$$ (63)

Applying the same method once again to (1+1) dimensional reduced PDE (63), reveals infinitesimals expressed as

$$\begin{array}{c} \xi_{r}=\frac{r}{2}c_{1}+c_{3},\\ \xi_{s}=c_{1}s+c_{2},\\ \eta_{g}=-\frac{g}{2}c_{1}. \end{array}$$ (64)

**Case 1.1.1**: Setting *c*_3_ = 1 and all other constants to zero, the characteristic form for (64) undergoes as

$$\frac{dg}{0}=\frac{dr}{1}=\frac{ds}{0},$$

and leaves the invariants (λ) = (*s*) by the transformation *g*(*r*, *s*) = *h*(λ). Then Eq (63) reduces to *h*′′ = 0, having solution

$$\begin{array}{c} h\left(\lambda \right)=c_{1}\lambda +c_{2}. \end{array}$$ (65)

Then we have

$$\begin{array}{c} g\left(r,s\right)=c_{1}s+c_{2}, \end{array}$$ (66)

$$\begin{array}{c} f\left(l,m,n\right)=c_{1}n+c_{2}. \end{array}$$ (67)

In conclusion, this case yields the following invariant solution for the 3D-EKP (2)

$$\begin{array}{c} U\left(x,y,z,t\right)=c_{1}z+c_{2}. \end{array}$$ (68)

**Case 1.1.2**: Setting *c*_2_ = 1 and all other constants to zero, the characteristic form for (64) undergoes as

$$\frac{dg}{0}=\frac{dr}{0}=\frac{ds}{1},$$

and leaves the invariants (λ) = (*r*) by the transformation *g*(*r*, *s*) = *h*(λ). Then Eq (63) reduces to following ODE,

$$\begin{array}{c} \left(h^{2}+2h^{\prime }\right)\left(hh^{\prime }+h^{\prime \prime }\right)=0. \end{array}$$ (69)

If *h*^2^ + 2*h*′ = 0, the resultant solution to the aforementioned ODE is as follows

$$\begin{array}{c} h\left(\lambda \right)=\frac{2}{2c_{1}+\lambda }, \end{array}$$ (70)

so the relation in variables (*r*, *s*) becomes

$$\begin{array}{c} g\left(r,s\right)=\frac{2}{2c_{1}+r}, \end{array}$$ (71)

and resulting back to the invariants (*l*, *m*, *n*)

$$\begin{array}{c} f\left(l,m,n\right)=\frac{2}{2c_{1}+l}\cdot \end{array}$$ (72)

In conclusion, this case yields the following invariant solution for the 3D-EKP (2)

$$\begin{array}{c} U\left(x,y,z,t\right)=\frac{2}{2c_{1}+x}\cdot \end{array}$$ (73)

If *h*^2^ + 2*h*′ ≠ 0 then *hh*′ + *h*′′ = 0, this implies,

$$\begin{array}{c} h\left(\lambda \right)=\frac{\sqrt{2}}{c_{1}}\;\text{tanh}\;(\frac{c_{2}+\lambda }{\sqrt{2}c_{1}}), \end{array}$$ (74)

$$\begin{array}{c} g\left(r,s\right)=\frac{\sqrt{2}}{c_{1}}\;\text{tanh}\;(\frac{c_{2}+r}{\sqrt{2}c_{1}}), \end{array}$$ (75)

$$\begin{array}{c} f\left(l,m,n\right)=\frac{\sqrt{2}}{c_{1}}\;\text{tanh}\;(\frac{c_{2}+l}{\sqrt{2}c_{1}}). \end{array}$$ (76)

In conclusion, this case yields the following invariant solution for the 3D-EKP (2)

$$\begin{array}{c} U\left(x,y,z,t\right)=\frac{\sqrt{2}}{c_{1}}\;\text{tanh}\;(\frac{c_{2}+x}{\sqrt{2}c_{1}}). \end{array}$$ (77)

**Case 1.2**: Setting *c*_4_ = 1 and all other constants to zero, the characteristic form for (62) undergoes as

$$\frac{df}{0}=\frac{dl}{1}=\frac{dm}{0}=\frac{dn}{0},$$

and leaves the invariants (*r*, *s*) = (*m*, *n*) by the transformation *f*(*l*, *m*, *n*) = *g*(*r*, *s*). Then Eq (61) reduces to (1+1) dimensional nonlinear PDE given by

$$\begin{array}{c} g_{ss}+g_{rr}=0. \end{array}$$ (78)

Applying the same method once again to (1+1) dimensional reduced PDE (78), reveals infinitesimals expressed as

$$\begin{array}{c} \xi_{r}=if_{5}\left(s-ir\right)-if_{6}\left(s+ir\right)+c_{2},\\ \xi_{s}=f_{5}\left(s-ir\right)+f_{6}\left(s+ir\right),\\ \eta_{g}=c_{1}g+f_{3}\left(s-ir\right)+f_{4}\left(s+ir\right). \end{array}$$ (79)

**Case 1.2.1**: Setting *c*_1_ = *c*_2_ = 1 and all other constants to zero, the characteristic form for (79) undergoes as

$$\frac{dg}{g}=\frac{dr}{1}=\frac{ds}{0},$$

and leaves the invariants (*g*, *s*) = (*e*^*r*^*h*(λ), λ) by the transformation *g*(*r*, *s*) = *h*(λ). Then Eq (78) reduces to the linear ODE *h*′′ + *h* = 0, having solution

$$\begin{array}{c} h\left(\lambda \right)=c_{1}\;\text{sin}\;\left(\lambda \right)+c_{2}\;\text{cos}\;\left(\lambda \right), \end{array}$$ (80)

$$\begin{array}{c} g\left(r,s\right)=\left(c_{1}\;\text{sin}\;\left(s\right)+c_{2}\;\text{cos}\;\left(s\right)\right)e^{r}. \end{array}$$ (81)

In the form of invariants (*l*, *m*, *n*), we get

$$\begin{array}{c} f\left(l,m,n\right)=\left(c_{1}\;\text{sin}\;\left(n\right)+c_{2}\;\text{cos}\;\left(n\right)\right)e^{m}. \end{array}$$ (82)

In conclusion, this case yields the following invariant solution for the 3D-EKP (2)

$$\begin{array}{c} U\left(x,y,z,t\right)=\left(c_{1}\;\text{sin}\;\left(z\right)+c_{2}\;\text{cos}\;\left(z\right)\right)e^{y}. \end{array}$$ (83)

**Case 2**: Ω_13_ = 〈*B*_3_〉.

The characteristic form for the vector field $B_{3}=\frac{\partial }{\partial y}$ can be formulated as

$$\frac{dU}{0}=\frac{dx}{0}=\frac{dy}{1}=\frac{dz}{0}=\frac{dt}{0},$$

and leaves the invariants (*l*, *m*, *n*) = (*t*, *x*, *z*) by the transformation *U*(*x*, *y*, *z*, *t*) = *f*(*l*, *m*, *n*). Then Eq (2) reduces to (2+1) dimensional nonlinear PDE given by

$$\begin{array}{c} \left(3f^{2}+6f_{m}\right)f_{mm}+3f^{3}f_{m}+6f{f_{m}}^{2}+2f_{nn}+2f_{lm}=0. \end{array}$$ (84)

Once more, the Lie symmetry method is applied to PDE (84), leading to the emergence of novel infinitesimals

$$\begin{array}{cc} \xi_{l} & =\frac{3}{2}c_{2}l+c_{4},\\ \xi_{m} & =\frac{c_{2}}{2}m-\frac{c_{1}}{2}n+c_{5},\\ \xi_{n} & =c_{1}l+c_{2}n+c_{3},\\ \eta_{f} & =-\frac{f}{2}c_{2}. \end{array}$$ (85)

**Case 2.1**: Setting *c*_4_ = *c*_5_ = 1 and all other constants to zero, the characteristic form for (85) undergoes as

$$\frac{df}{0}=\frac{dl}{1}=\frac{dm}{1}=\frac{dn}{0},$$

and leaves the invariants (*r*, *s*) = (*n*, −*l* + *m*) by the transformation *f*(*l*, *m*, *n*) = *g*(*r*, *s*). Then Eq (84) reduces to (1+1) dimensional nonlinear PDE given by

$$\begin{array}{c} \left(3g^{2}+6g_{s}-2\right)g_{ss}+3g^{3}g_{s}+6g{g_{s}}^{2}+2g_{rr}=0. \end{array}$$ (86)

Once more, the Lie symmetry method is applied to PDE (86), leading to the emergence of novel infinitesimals

$$\begin{array}{c} \begin{array}{c} \xi_{r}=c_{1},\\ \xi_{s}=c_{2},\\ \eta_{g}=0. \end{array} \end{array}$$ (87)

**Case 2.1.1**: Setting *c*_1_ = *c*_2_ = 1 and all other constants to zero, the characteristic form for (87) undergoes as

$$\frac{dg}{0}=\frac{dr}{1}=\frac{ds}{1},$$

and leaves the invariants (λ) = (*s* − *r*) by the transformation *g*(*r*, *s*) = *h*(λ). Then Eq (86) reduces to following ODE

$$\begin{array}{c} \left(h^{2}+2h^{\prime }\right)\left(hh^{\prime }+h^{\prime \prime }\right)=0. \end{array}$$ (88)

If *h*^2^ + 2*h*′ = 0, this implies,

$$\begin{array}{c} h\left(\lambda \right)=\frac{2}{2c_{1}+\lambda }, \end{array}$$ (89)

$$\begin{array}{c} g\left(r,s\right)=\frac{2}{2c_{1}+s-r}, \end{array}$$ (90)

$$\begin{array}{c} f\left(l,m,n\right)=\frac{2}{2c_{1}+m-l-n}\cdot \end{array}$$ (91)

In conclusion, this case yields the following invariant solution for the 3D-EKP (2)

$$\begin{array}{c} U\left(x,y,z,t\right)=\frac{2}{2c_{1}+x-z-t}\cdot \end{array}$$ (92)

If *h*^2^ + 2*h*′ ≠ 0 then *hh*′ + *h*′′ = 0, this implies,

$$\begin{array}{c} h\left(\lambda \right)=\frac{\sqrt{2}}{c_{1}}\;\text{tanh}\;(\frac{c_{2}+\lambda }{\sqrt{2}c_{1}}), \end{array}$$ (93)

$$\begin{array}{c} g\left(r,s\right)=\frac{\sqrt{2}}{c_{1}}\;\text{tanh}\;(\frac{c_{2}+s-r}{\sqrt{2}c_{1}}), \end{array}$$ (94)

$$\begin{array}{c} f\left(l,m,n\right)=\frac{\sqrt{2}}{c_{1}}\;\text{tanh}\;(\frac{c_{2}+m-l-n}{\sqrt{2}c_{1}})\cdot \end{array}$$ (95)

In conclusion, this case yields the following invariant solution for the 3D-EKP (2)

$$\begin{array}{c} U\left(x,y,z,t\right)=\frac{\sqrt{2}}{c_{1}}\;\text{tanh}\;(\frac{c_{2}+x-z-t}{\sqrt{2}c_{1}}). \end{array}$$ (96)

**Case 2.2**: Setting *c*_3_ = *c*_4_ = 1 and all other constants to zero, the characteristic form for (85) undergoes as

$$\frac{df}{0}=\frac{dl}{1}=\frac{dm}{0}=\frac{dn}{1},$$

and leaves the invariants (*r*, *s*) = (*m*, *n* − *l*) by the transformation *f*(*l*, *m*, *n*) = *g*(*r*, *s*). Then Eq (84) reduces to (1+1) dimensional nonlinear PDE given by

$$\begin{array}{c} \left(3g^{2}+6g_{r}\right)g_{rr}+3g^{3}g_{r}+6g{g_{r}}^{2}-2g_{rs}+2g_{ss}=0. \end{array}$$ (97)

Iteratively, the Lie symmetry method is implemented to analyze the PDE (86), yielding the discovery of new infinitesimals

$$\begin{array}{c} \xi_{r}=c_{1},\\ \xi_{s}=c_{2},\\ \eta_{g}=0. \end{array}$$ (98)

**Case 2.2.1**: Setting *c*_1_ = 1 and all other constants to zero, the characteristic form for (98) undergoes as

$$\frac{dg}{0}=\frac{dr}{1}=\frac{ds}{0},$$

and leaves the invariants (*s*) = (λ) by the transformation *g*(*r*, *s*) = *h*(λ). Then Eq (97) reduces to the linear ODE *h*′′ = 0, having solution

$$\begin{array}{c} h\left(\lambda \right)=c_{1}\lambda +c_{2}, \end{array}$$ (99)

$$\begin{array}{c} g\left(r,s\right)=c_{1}s+c_{2}. \end{array}$$ (100)

In the form of invariants (*l*, *m*, *n*), we get

$$\begin{array}{c} f\left(l,m,n\right)=c_{1}\left(-l+n\right)+c_{2}. \end{array}$$ (101)

In conclusion, this case yields the following invariant solution for the 3D-EKP (2)

$$\begin{array}{c} U\left(x,y,z,t\right)=c_{1}\left(z-t\right)+c_{2}. \end{array}$$ (102)

**Case 2.3**: Setting *c*_3_ = 1 and all other constants to zero, the characteristic form for (85) undergoes as

$$\frac{df}{0}=\frac{dl}{0}=\frac{dm}{0}=\frac{dn}{1},$$

and leaves the invariants (*r*, *s*) = (*l*, *m*) by the transformation *f*(*l*, *m*, *n*) = *g*(*r*, *s*). Then Eq (84) reduces to (1+1) dimensional PDE given by

$$\begin{array}{c} \left(3g^{2}+6g_{s}\right)g_{ss}+3g^{3}g_{s}+6g{g_{s}}^{2}+2g_{rs}=0. \end{array}$$ (103)

Iteratively, the Lie symmetry method is implemented to analyze the PDE (103), yielding the discovery of new infinitesimals.

$$\begin{array}{c} \xi_{r}=c_{1}r+c_{2},\\ \xi_{s}=\frac{c_{1}}{3}s+c_{3},\\ \eta_{g}=-\frac{c_{1}}{3}g. \end{array}$$ (104)

**Case 2.3.1**: Setting *c*_2_ = 1 and all other constants to zero, the characteristic form for (104) undergoes as

$$\frac{dg}{0}=\frac{dr}{1}=\frac{ds}{0},$$

and leaves the invariants (λ) = (*s*) by the transformation *g*(*r*, *s*) = *h*(λ). Then Eq (103) reduces to following ODE

$$\begin{array}{c} \left(h^{2}+2h^{\prime }\right)\left(hh^{\prime }+h^{\prime \prime }\right)=0. \end{array}$$ (105)

If *h*^2^ + 2*h*′ = 0, this implies,

$$\begin{array}{c} h\left(\lambda \right)=\frac{2}{2c_{1}+\lambda }, \end{array}$$ (106)

$$\begin{array}{c} g\left(r,s\right)=\frac{2}{2c_{1}+s}, \end{array}$$ (107)

$$\begin{array}{c} f\left(l,m,n\right)=\frac{2}{2c_{1}+m}\cdot \end{array}$$ (108)

In conclusion, this case yields the following invariant solution for the 3D-EKP (2)

$$\begin{array}{c} U\left(x,y,z,t\right)=\frac{2}{2c_{1}+x}\cdot \end{array}$$ (109)

If *h*^2^ + 2*h*′ ≠ 0 then *hh*′ + *h*′′ = 0, this implies,

$$\begin{array}{c} h\left(\lambda \right)=\frac{\sqrt{2}}{c_{1}}\;\text{tanh}\;(\frac{c_{2}+\lambda }{\sqrt{2}c_{1}}), \end{array}$$ (110)

$$\begin{array}{c} g\left(r,s\right)=\frac{\sqrt{2}}{c_{1}}\;\text{tanh}\;(\frac{c_{2}+s}{\sqrt{2}c_{1}}), \end{array}$$ (111)

$$\begin{array}{c} f\left(l,m,n\right)=\frac{\sqrt{2}}{c_{1}}\;\text{tanh}\;(\frac{c_{2}+m}{\sqrt{2}c_{1}})\cdot \end{array}$$ (112)

In conclusion, this case yields the following invariant solution for the 3D-EKP (2)

$$\begin{array}{c} U\left(x,y,z,t\right)=\frac{\sqrt{2}}{c_{1}}\;\text{tanh}\;(\frac{c_{2}+x}{\sqrt{2}c_{1}}). \end{array}$$ (113)

**Case 3**: Ω_12_ = 〈*B*_4_〉.

The characteristic form for the vector field $B_{4}=\frac{\partial }{\partial z}$ can be formulated as

$$\frac{dU}{0}=\frac{dx}{0}=\frac{dy}{0}=\frac{dz}{1}=\frac{dt}{0},$$

and leaves the invariants (*l*, *m*, *n*) = (*t*, *x*, *y*) by the transformation *U*(*x*, *y*, *z*, *t*) = *f*(*l*, *m*, *n*). Then Eq (2) reduces to (2+1) dimensional nonlinear PDE given by

$$\begin{array}{c} \left(3f^{2}+6f_{m}\right)f_{mm}+3f^{3}f_{m}+6f{f_{m}}^{2}+2f_{nn}+2f_{lm}=0. \end{array}$$ (114)

Iteratively, the Lie symmetry method is implemented to analyze the PDE (114), yielding the discovery of new infinitesimals.

$$\begin{array}{c} \begin{array}{cc} \xi_{l} & =\frac{3}{2}c_{2}l+c_{4},\\ \xi_{m} & =\frac{c_{2}}{2}m-\frac{c_{1}}{2}n+c_{5},\\ \xi_{n} & =c_{1}l+c_{2}n+c_{3},\\ \eta_{f} & =-\frac{f}{2}c_{2}. \end{array} \end{array}$$ (115)

**Case 3.1**: Setting *c*_4_ = 1 and all other constants to zero, the characteristic form for (115) undergoes as

$$\frac{df}{0}=\frac{dl}{1}=\frac{dm}{0}=\frac{dn}{0},$$

and leaves the invariants (*r*, *s*) = (*m*, *n*) by the transformation *f*(*l*, *m*, *n*) = *g*(*r*, *s*). Then Eq (114) reduces to (1+1) dimensional nonlinear PDE given by

$$\begin{array}{c} \left(3g^{2}+6g_{r}\right)g_{rr}+3g^{3}g_{r}+6g{g_{r}}^{2}+2g_{ss}=0. \end{array}$$ (116)

Iteratively, the Lie symmetry method is implemented to analyze the PDE (116), yielding the discovery of new infinitesimals.

$$\begin{array}{c} \begin{array}{cc} \xi_{r} & =\frac{r}{2}c_{1}+c_{3},\\ \xi_{s} & =c_{1}s+c_{2},\\ \eta_{g} & =-\frac{c_{1}}{2}g. \end{array} \end{array}$$ (117)

**Case 3.1.1**: Setting *c*_3_ = 1 and all other constants to zero, the characteristic form for (117) undergoes as

$$\frac{dg}{0}=\frac{dr}{1}=\frac{ds}{0},$$

and leaves the invariants (λ) = (*s*) by the transformation *g*(*r*, *s*) = *h*(λ). Then Eq (116) reduces to *h*′′ = 0, having solution

$$\begin{array}{c} h\left(\lambda \right)=c_{1}\lambda +c_{2}, \end{array}$$ (118)

$$\begin{array}{c} g\left(r,s\right)=c_{1}s+c_{2}. \end{array}$$ (119)

In the form invariants (*l*, *m*, *n*), we get

$$\begin{array}{c} f\left(l,m,n\right)=c_{1}n+c_{2}. \end{array}$$ (120)

In conclusion, this case yields the following invariant solution for the 3D-EKP (2)

$$\begin{array}{c} U\left(x,y,z,t\right)=c_{1}y+c_{2}. \end{array}$$ (121)

**Case 3.1.2**: Setting *c*_2_ = *c*_3_ = 1 and all other constants to zero, the characteristic form for (98) undergoes

$$\frac{dg}{0}=\frac{dr}{1}=\frac{ds}{1},$$

and leaves the invariants (λ) = (*s* − *r*) by the transformation *g*(*r*, *s*) = *h*(λ). Then Eq (116) reduces to following ODE

$$\begin{array}{c} \left(3h^{2}-6h^{\prime }+2\right)h^{\prime \prime }-3h^{3}h^{\prime }+6h{h^{\prime }}^{2}=0. \end{array}$$ (122)

Infinitesimals of ODE (122) are given by,

$$\begin{array}{c} \xi_{\lambda }=1,\;\eta_{h}=0. \end{array}$$ (123)

By the utilization of infinitesimals (123), the solution of ODE (122) becomes,

$$\begin{array}{c} \int \frac{dh}{\frac{h^{2}}{2}+\frac{1}{3}+\frac{1}{3}\sqrt{3h^{2}+3c_{1}+1}}-\lambda -c_{2}=0. \end{array}$$ (124)

In conclusion, this case yields the following invariant solution for the 3D-EKP (2)

$$\begin{array}{c} \int \frac{dU}{\frac{U^{2}}{2}+\frac{1}{3}+\frac{1}{3}\sqrt{3U^{2}+3c_{1}+1}}-\left(y-x\right)-c_{2}=0. \end{array}$$ (125)

**Case 3.2**: Setting *c*_3_ = *c*_4_ = 1 and all other constants to zero, the characteristic form for (115) undergoes as

$$\frac{df}{0}=\frac{dl}{1}=\frac{dm}{0}=\frac{dn}{1},$$

and leaves the invariants (*r*, *s*) = (*m*, *n* − *l*) by the transformation *f*(*l*, *m*, *n*) = *g*(*r*, *s*). Then Eq (114) reduces to (1+1) dimensional nonlinear PDE given by

$$\begin{array}{c} \left(3g^{2}+6g_{r}\right)g_{rr}+3g^{3}g_{r}+6g{g_{r}}^{2}-2g_{rs}+2g_{ss}=0. \end{array}$$ (126)

Iteratively, the Lie symmetry method is implemented to analyze the PDE (126), yielding the discovery of new infinitesimals.

$$\begin{array}{c} \xi_{r}=c_{1},\\ \xi_{s}=c_{2},\\ \eta_{g}=0. \end{array}$$ (127)

**Case 3.2.1**: Setting *c*_1_ = 1 and all other constants to zero, the characteristic form for (127) undergoes as

$$\frac{dg}{0}=\frac{dr}{1}=\frac{ds}{0},$$

and leaves the invariants (*s*) = (λ) by the transformation *g*(*r*, *s*) = *h*(λ). Then Eq (126) reduces to the linear ODE *h*′′ = 0, having solution this implies,

$$\begin{array}{c} h\left(\lambda \right)=c_{1}\lambda +c_{2}, \end{array}$$ (128)

$$\begin{array}{c} g\left(r,s\right)=c_{1}s+c_{2}, \end{array}$$ (129)

$$\begin{array}{c} f\left(l,m,n\right)=c_{1}n+c_{2}. \end{array}$$ (130)

In conclusion, this case yields the following invariant solution for the 3D-EKP (2)

$$\begin{array}{c} U\left(x,y,z,t\right)=c_{1}\left(y-t\right)+c_{2}. \end{array}$$ (131)

**Case 4**: Ω_14_ = 〈*B*_2_〉.

The characteristic form for the vector field $B_{2}=\frac{\partial }{\partial x}$ can be formulated as

$$\frac{dU}{0}=\frac{dx}{1}=\frac{dy}{0}=\frac{dz}{0}=\frac{dt}{0},$$

and leaves the invariants (*l*, *m*, *n*) = (*t*, *y*, *z*) by the transformation *U*(*x*, *y*, *z*, *t*) = *f*(*l*, *m*, *n*, *s*). Then Eq (2) reduces to (2+1) dimensional nonlinear PDE given by

$$\begin{array}{c} f_{mm}+f_{nn}=0. \end{array}$$ (132)

This implies,

$$\begin{array}{c} f\left(l,m,n\right)=F_{1}\left(l,n-im\right)+F_{2}\left(l,n+im\right). \end{array}$$ (133)

In conclusion, this case yields the following invariant solution for the 3D-EKP (2)

$$\begin{array}{c} U\left(x,y,z,t\right)=F_{1}\left(t,z-iy\right)+F_{2}\left(t,z+iy\right). \end{array}$$ (134)

## 4 Local conservation laws for Eq (2)

For a scalar PDE,

$$\begin{array}{c} H(t,x,y,z,U,U_{t},U_{x},U_{y},U_{z},\ldots )=0, \end{array}$$ (135)

a local conservation law for *U*(*x*, *y*, *z*, *t*) is the divergence equation [38, 39]

$$\begin{array}{c} D_{t}T^{t}+D_{x}K^{x}+D_{y}K^{y}+D_{z}K^{z}=0, \end{array}$$ (136)

continuing within the solution space E of the given PDE, wherein $T^{t}$ signifies the conserved density, and $K=(K^{x},K^{y},K^{z})$ constitutes the spatial flux vector. The dependence of K on *t*, *x*, *y*, *z*, *U*, and its derivatives is acknowledged. The pair (T,K) is employed to denote the conserved current.

Conservation laws for the PDE H=0 are exclusively derived from non-trivial multipliers. This derivation establishes a one-to-one mapping with non-trivial conserved currents ${\left(T,K\right)|}_{E}$, excluding the trivial ones. This correspondence is contingent on the presence of non-zero multipliers ${\Lambda |}_{E}$, satisfying $\Lambda H=D_{t}T^{t}+D_{x}K^{x}+D_{y}K^{y}+D_{z}K^{z}$ and maintaining it as an identity.

For 3D-EKP Eq (2), the characteristic form for the conservation laws is written as

$$\begin{array}{c} D_{t}T^{t}+D_{x}K^{x}+D_{y}K^{y}+D_{z}K^{z}=(U_{tx}+\frac{3}{2}U^{3}U_{x}+\frac{3}{2}U^{2}U_{xx}+3UU_{x}^{2}+3U_{x}U_{xx}+U_{yy}+U_{zz})\Lambda . \end{array}$$ (137)

The multipliers Λ(*t*, *x*, *y*, *z*, *U*) are determined through the fulfillment of the divergence condition

$$\begin{array}{c} \frac{\delta }{\delta U}((U_{tx}+\frac{3}{2}U^{3}U_{x}+\frac{3}{2}U^{2}U_{xx}+3UU_{x}^{2}+3U_{x}U_{xx}+U_{yy}+U_{zz})\Lambda )=0, \end{array}$$ (138)

where $\frac{\delta }{\delta U}$ denotes the Euler operator relative to *U*. The imposition of the divergence condition onto the multipliers Λ, coupled with their decomposition relative to the derivatives of *U*(*x*, *y*, *z*, *t*), engenders an overdetermined system. This system is amenable to direct resolution, culminating in the articulation of the subsequent proposition:

**Proposition 1** *The low-order (characteristics) multipliers, permissible within the context of the (3+1)-dimensional EKP* Eq (2), *are elucidated in a subsequent manner*

$$\begin{array}{c} \Lambda_{1}=1,\\ \Lambda_{2}=t,\\ \Lambda_{3}=z,\\ \Lambda_{4}=y,\\ \Lambda_{5}=t^{2},\\ \Lambda_{6}=zt,\\ \Lambda_{7}=yt,\\ \Lambda_{8}=yz,\\ \Lambda_{9}=y^{2}-z^{2}. \end{array}$$ (139)

These multipliers lead to the development of noteworthy low-order conservation laws, which can be found below:

**Theorem 1** *The local conservation laws corresponding to the (3+1)-dimensional EKP* Eq (2) *are given by*

$$\begin{array}{c} \begin{array}{cc} T_{1}^{t} & =U_{x},\\ K_{1}^{x} & =\frac{3}{8}{\left(U^{2}+2U_{x}\right)}^{2},\\ K_{1}^{y} & =U_{y},\\ K_{1}^{z} & =U_{z}. \end{array} \end{array}$$ (140)

$$\begin{array}{c} \begin{array}{cc} T_{2}^{t} & =tU_{x},\\ K_{2}^{x} & =\frac{3}{8}t{\left(U^{2}+2U_{x}\right)}^{2},\\ K_{2}^{y} & =tU_{y},\\ K_{2}^{z} & =tU_{z}. \end{array} \end{array}$$ (141)

$$\begin{array}{c} \begin{array}{cc} T_{3}^{t} & =zU_{x},\\ K_{3}^{x} & =\frac{3}{8}z{\left(U^{2}+2U_{x}\right)}^{2},\\ K_{3}^{y} & =zU_{y}-yU_{z},\\ K_{3}^{z} & =yU_{y}+zU_{z}. \end{array} \end{array}$$ (142)

$$\begin{array}{c} \begin{array}{cc} T_{4}^{t} & =yU_{x},\\ K_{4}^{x} & =\frac{3}{8}y{\left(U^{2}+2U_{x}\right)}^{2}-xU_{y},\\ K_{4}^{y} & =xU_{x}+yU_{y},\\ K_{4}^{z} & =yU_{z}. \end{array} \end{array}$$ (143)

$$\begin{array}{c} \begin{array}{cc} T_{5}^{t} & =t^{2}U_{x},\\ K_{5}^{x} & =\frac{3}{8}t({\left(U^{2}+2U_{x}\right)}^{2}t-\frac{16}{3}U),\\ K_{5}^{y} & =t^{2}U_{y},\\ K_{5}^{z} & =t^{2}U_{z}. \end{array} \end{array}$$ (144)

$$\begin{array}{c} \begin{array}{cc} T_{6}^{t} & =ztU_{x},\\ K_{6}^{x} & =\frac{3}{8}z({\left(U^{2}+2U_{x}\right)}^{2}t-\frac{8}{3}U),\\ K_{6}^{y} & =t(zU_{y}-yU_{z}),\\ K_{6}^{z} & =t(yU_{y}+zU_{z}). \end{array} \end{array}$$ (145)

$$\begin{array}{c} \begin{array}{cc} T_{7}^{t} & =ytU_{x},\\ K_{7}^{x} & =\frac{t}{8}(3y{\left(U^{2}+2U_{x}\right)}^{2}-8xU_{y})-yU,\\ K_{7}^{y} & =t(xU_{x}+yU_{y}),\\ K_{7}^{z} & =ytU_{z}. \end{array} \end{array}$$ (146)

$$\begin{array}{c} \begin{array}{cc} T_{8}^{t} & =yzU_{x},\\ K_{8}^{x} & =\frac{3}{8}z({\left(U^{2}+2U_{x}\right)}^{2}y-\frac{8}{3}xU_{y}),\\ K_{8}^{y} & =z\left(xU_{x}+yU_{y}\right)-\frac{y^{2}}{2}U_{z},\\ K_{8}^{z} & =yzU_{z}+\frac{y^{2}}{2}U_{y}. \end{array} \end{array}$$ (147)

$$\begin{array}{c} \begin{array}{cc} T_{9}^{t} & =y^{2}U_{x}-z^{2}U_{x},\\ K_{9}^{x} & =\frac{3}{8}\left(y-z\right)\left(y+z\right){\left(U^{2}+2U_{x}\right)}^{2},\\ K_{9}^{y} & =\left(y^{2}-z^{2}\right)U_{y}+2yzU_{z},\\ K_{9}^{z} & =\left(y^{2}-z^{2}\right)U_{z}-2yzU_{y}. \end{array} \end{array}$$ (148)

## 5 Graphical interpretation of invariant solutions

**Fig 1:**
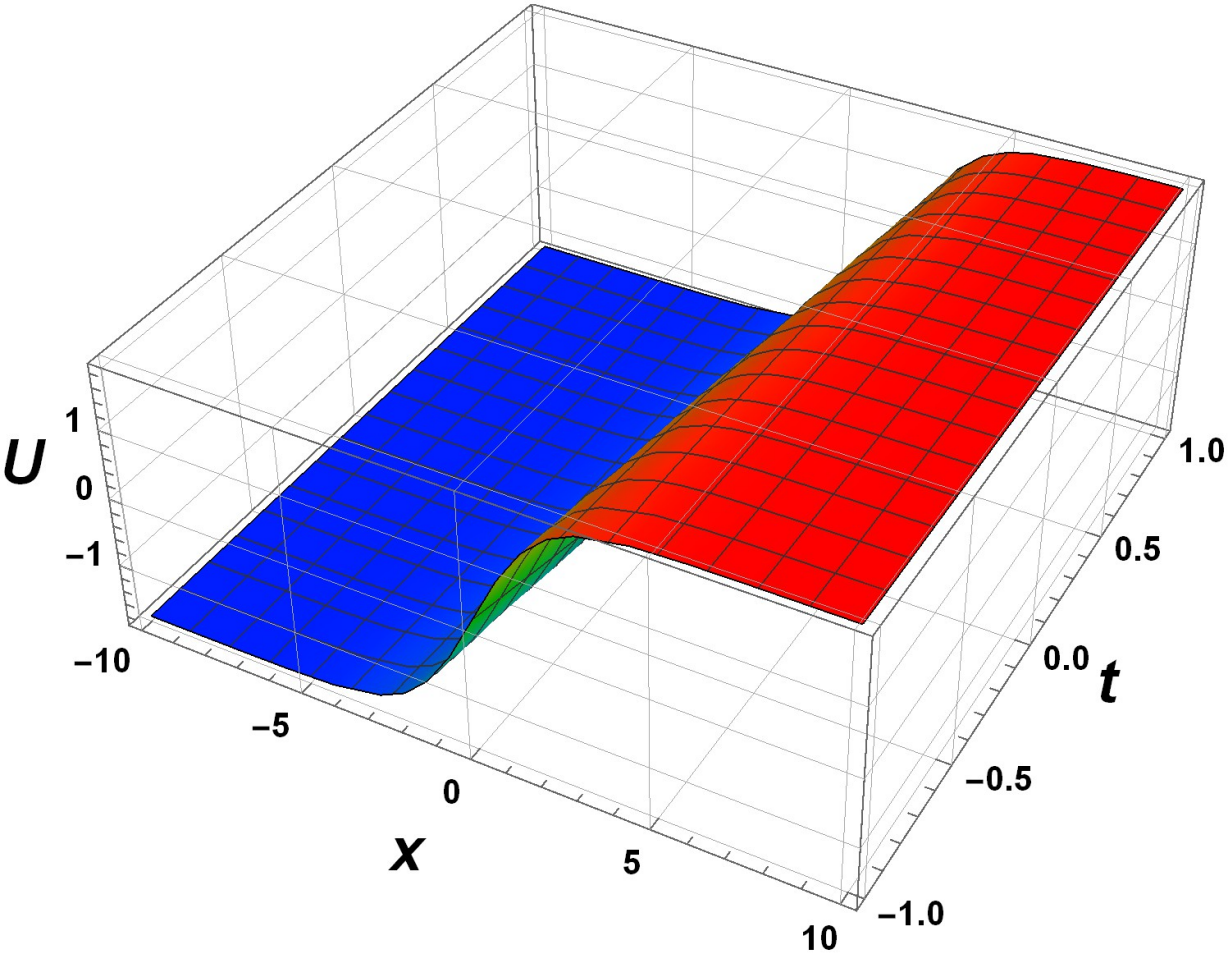
Surface dynamics of the 3D-EKP Eq (2) by the invariant solution (92) with *c*_1_ = *c*_2_ = 1.

**Fig 2:**
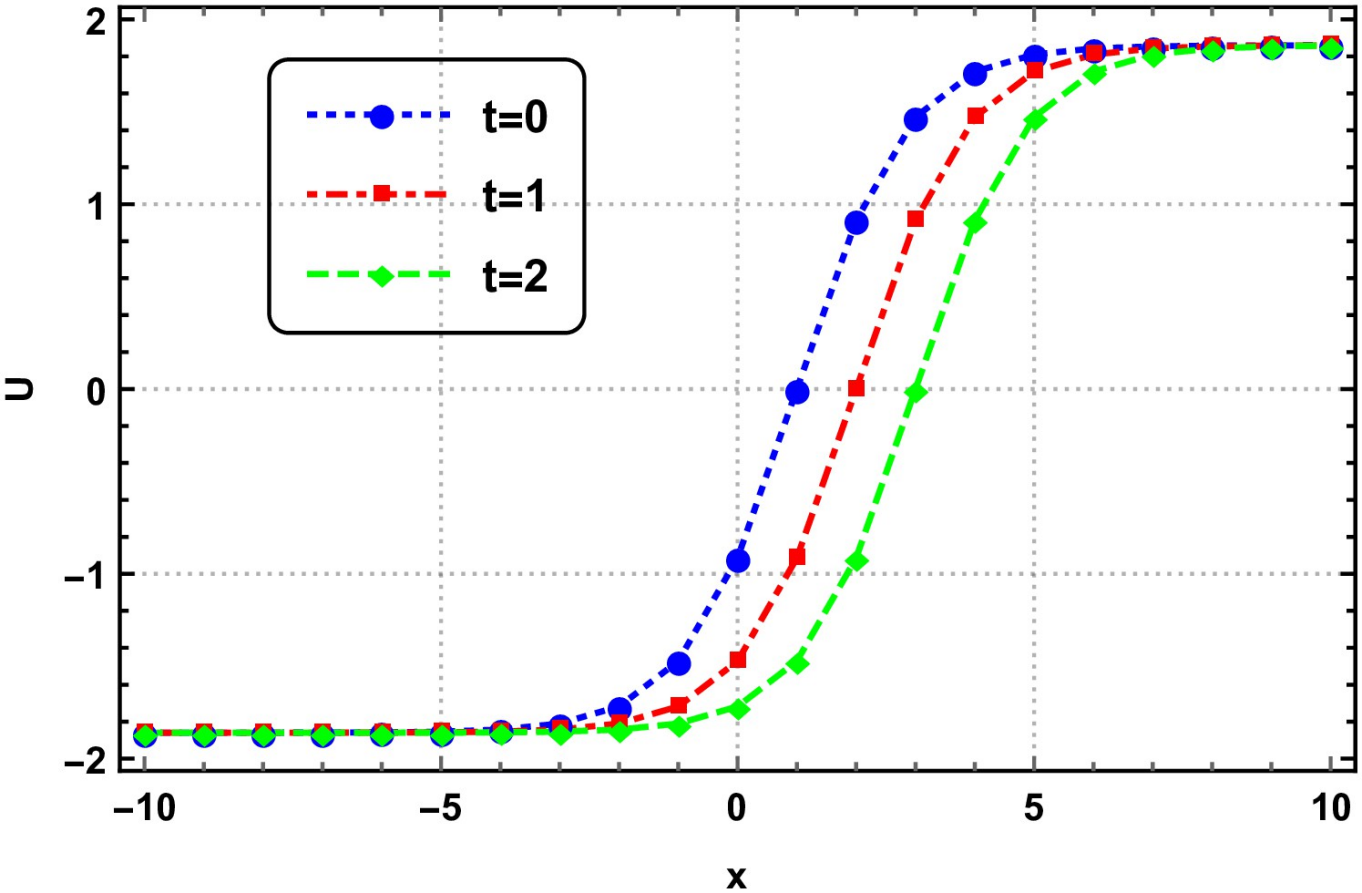
Surface dynamics of the 3D-EKP Eq (2) by the invariant solution (92) with *c*_1_ = *c*_2_ = 1.

**Fig 3:**
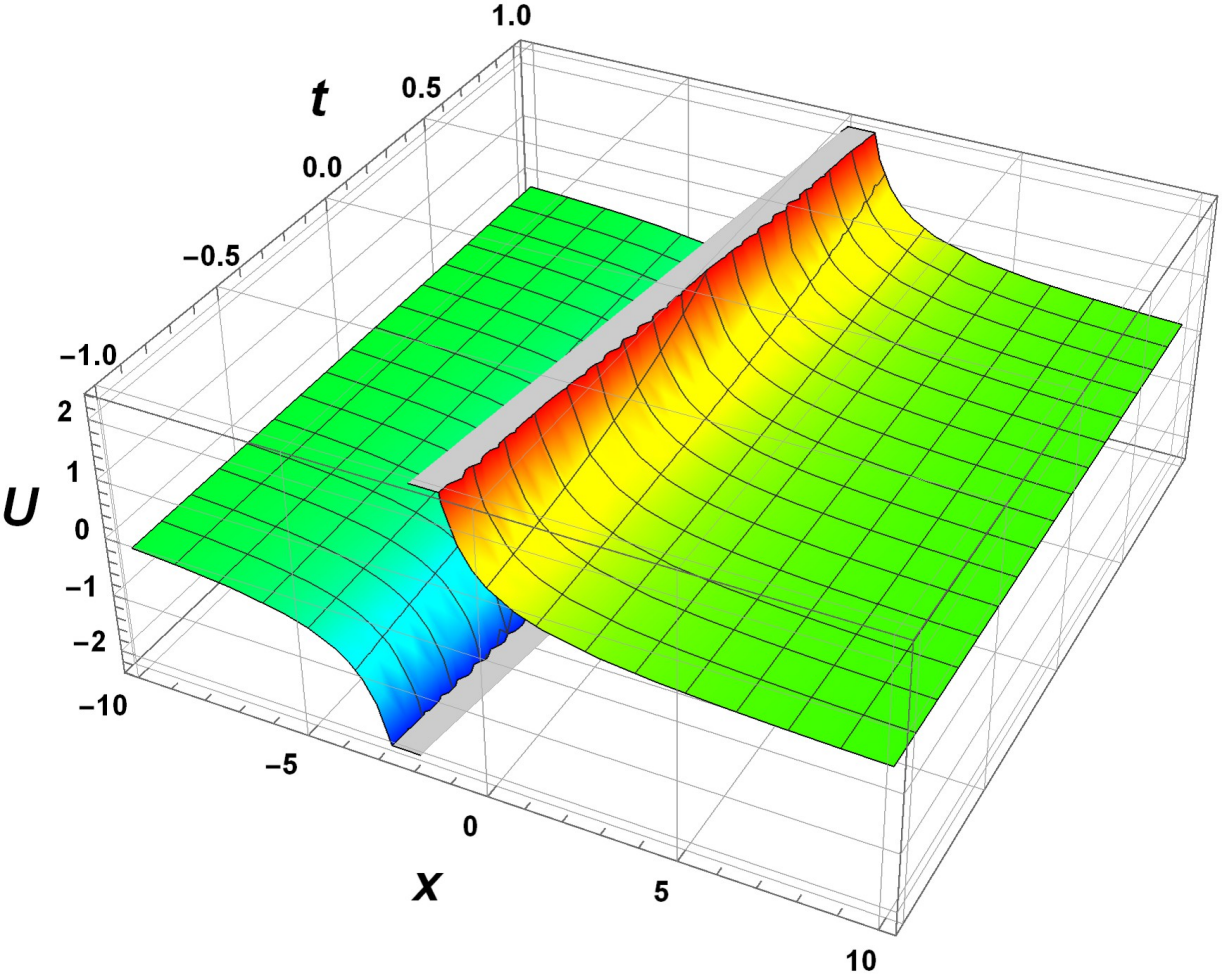
Surface dynamics of the 3D-EKP Eq (2) by the invariant solution (96) with *c*_1_ = *c*_2_ = 1.

**Fig 4:**
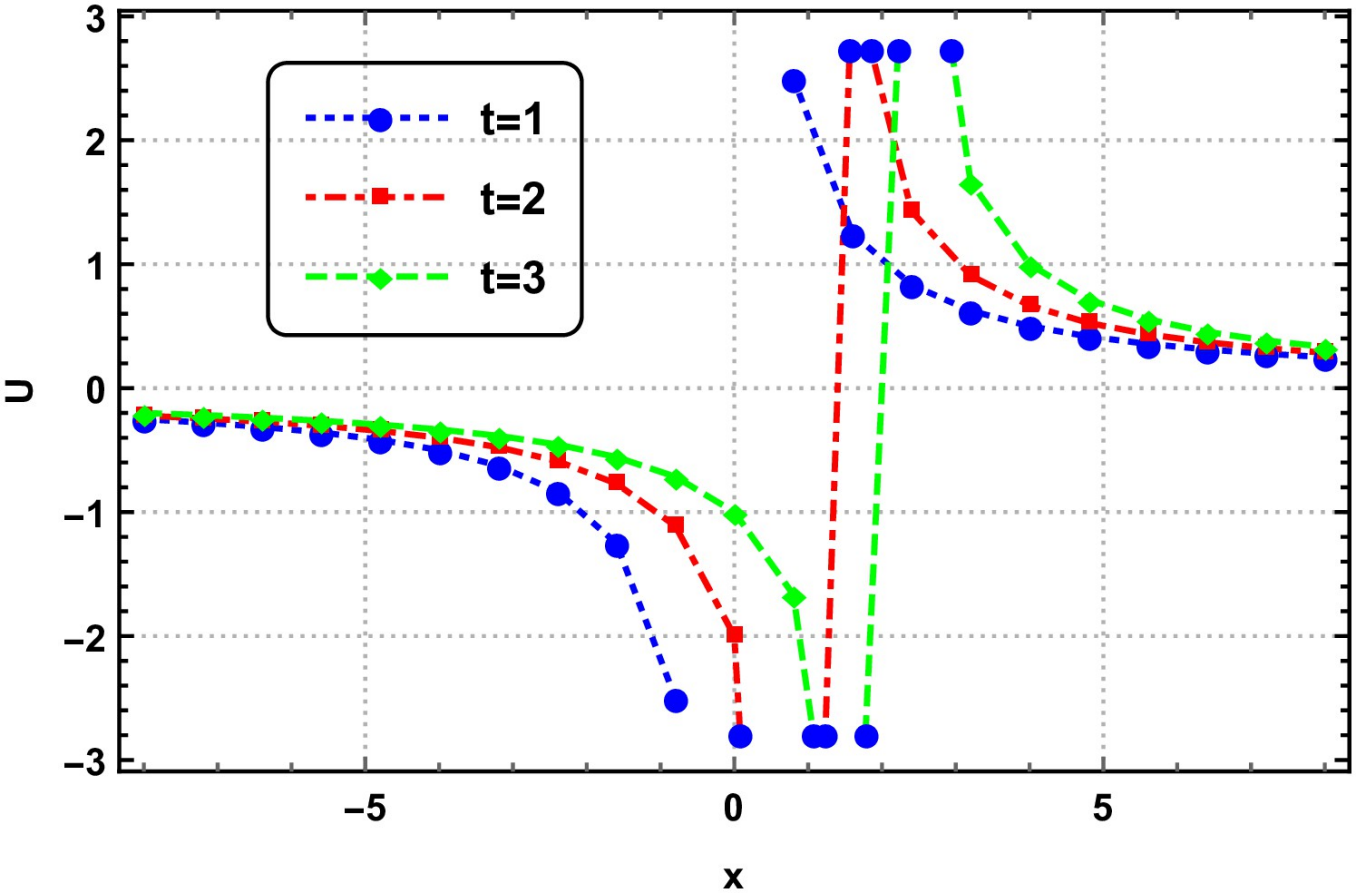
Surface dynamics of the 3D-EKP Eq (2) by the invariant solution (96) with *c*_1_ = *c*_2_ = 1.

Graphical interpretation is crucial when analyzing solutions of differential equations, particularly NLPDEs. This approach offers several key benefits. Firstly, graphical representations visually convey the behavior of the solution, allowing for intuitive understanding and insight into complex dynamics. Secondly, graphs aid in identifying qualitative features such as stability, periodicity, or the presence of critical points. Moreover, graphs provide a means to validate numerical approximations or experimental data against the expected behavior. Overall, graphical interpretation enhances the understanding, analysis, and visualization of solutions in NLPDEs, enabling deeper insights and facilitating scientific discoveries. Here we represent the interpretation in Figs 1–4.

## 6 Concluding remarks

In this investigation, we harnessed the potent technique of Lie group analysis to derive analytical solutions for the 3D-EKP Eq (2). Systematically applying this method allowed us to identify successive reductions and the associated Lie point symmetries of the considered equation. This analysis resulted in the derivation of an optimal system of subalgebras relevant to the equation. Lü *et al.* [27] previously derived this equation and reported eighteen classes of rational solutions, while Yu and Sun [28] constructed lump solutions rationally localized in all spatial directions for its two-dimensionally reduced cases using a generalized bilinear differential equation method. However, none of these solutions exhibit invariance under a symmetry generator. In contrast, our results encompass rational, polynomial, hyperbolic, and trigonometric solution structures that differ from the studies mentioned above due to their invariance under a specific symmetry generator, representing more general solution classes. This underscores the novelty of our findings. Additionally, we investigated local conservation laws using a direct approach first in the theory of the EKP Eq (2). These results affirm the efficacy of the Lie group method in handling NLPDEs. Through these efforts, we gained valuable insights into the mathematical properties and symmetries of the 3D-EKP Eq (2), enhancing our comprehension of its dynamics and laying the groundwork for further analysis and applications. Moving forward, our motivation extends to exploring soliton solutions for the considered equation.
